# Habitat preferences of Southern Ground-hornbills in the Kruger National Park: implications for future conservation measures

**DOI:** 10.1038/s41598-020-73236-4

**Published:** 2020-10-01

**Authors:** Leigh Combrink, Hendrik J. Combrink, André J. Botha, Colleen T. Downs

**Affiliations:** 1 Centre for Functional Biodiversity, School of Life Sciences, University of KwaZulu-Natal, P/Bag X01, Scottsville, Pietermaritzburg, 3209 South Africa; 2 The Endangered Wildlife Trust, P/Bag X11, Modderfontein, Johannesburg, 1645 South Africa; 3 Department of Biomedical Sciences, Oregon State University, Corvallis, OR USA; 4 Department of Integrative Biology, Oregon State University, Corvallis, OR USA

**Keywords:** Ecology, Behavioural ecology

## Abstract

Understanding how a species utilises its habitat, and the processes that give rise to its movements and patterns of space use, is critical for its conservation. Southern Ground-hornbills *Bucorvus leadbeateri* are listed as Endangered in South Africa, as a result of habitat loss and persecution. The National Species Recovery Plan lists reintroductions as a suitable conservation action, but highlights “understanding the exact habitat requirements of Southern Ground-hornbills” as a knowledge gap. In this study, we used tracking data from six Southern Ground-hornbill groups (a total of 37,060 GPS locations) in the Kruger National Park to investigate their seasonal home range differences and habitat preferences. We used first-passage time analysis to determine the scale at which Southern Ground-hornbills concentrate their foraging efforts and whether specific movement behaviours were linked to habitat types. We found marked differences in seasonal home ranges, with all groups showing a range contraction during the breeding season. Grassland and open woodland habitat types were used throughout the year in accordance with their availability within the territory, with grassland, open woodland and dense thicket being favoured habitats for foraging. Our habitat preference results, based on longitudinal GPS data, allowed us to determine ideal habitat ratios (grassland:open woodland:low shrubland of 1.00:6.10:0.09 ha) to assist with the selection of suitable reintroduction sites for Southern Ground-hornbills. With an increasing number of species being threatened with extinction, reintroductions into suitable habitats may be a useful conservation mitigation measure. However, our findings highlight the importance of a thorough understanding of a species’ movement and space use prior to the selection of areas for reintroduction to ensure the establishment and sustainability of these species at these sites.

## Introduction

With the advent of advances in satellite tracking technology and the accompanying improvements in analytical tools, the field of movement ecology has developed rapidly over the last decade^1–3^. To optimise fitness, an individual will move to exploit resources within their prescribed habitat, the availability of which changes in space and time^4^. How a species utilises its habitat and understanding the processes that give rise to its movement and pattern of space use, is paramount to its conservation^3,5–7^.

Movement patterns and space use determine species distributions and home ranges^2,8^. Most species have spatially heterogeneous home ranges, where resources are not evenly distributed in space or time^7^. In response to the local abundance of a particular resource, many species will alter their speed of movement or the tortuosity of their movement paths to take advantage of this opportunity^8^. Optimal foraging will result in animals conducting intensive searches in patches of high resource density and minimising time spent in lower resource density areas^7^. This particular movement pattern, termed an area-concentrated search, results in slow, tortuous paths within a particular resource patch and fast, direct paths between patches^8,9^. Another measure of movement is first-passage time, which is the time taken for a species to cross a circle with a given radius^9^, allowing researchers to determine both search effort and the spatial scales at which species concentrate this effort^9,10^.

The use of tracking technology has allowed researchers to link movement patterns and relocation data with global information systems (GIS) mapping, habitat and environmental variables, providing a useful way of determining the influence of habitat and environmental features on animal movement^6,11^. Understanding these patterns of movement and habitat use and relating these to environmental variables and habitat heterogeneity will assist in modelling and predicting species’ home ranges in different regions and determining their minimum habitat requirements^7^. This is particularly important in the conservation of endangered species.

Southern Ground-hornbills *Bucorvus leadbeateri* are large, terrestrial, carnivorous birds that inhabit savanna and bushveld habitats throughout much of Africa, south of the equator^12^. They are co-operative breeders, forming groups with an alpha breeding pair and up to nine helper birds, and they forage as a cohesive unit^12^. They are secondary cavity nesters, primarily occupying natural cavities in large trees, but occasionally nesting in cavities in cliffs or earth banks^13^. Females lay two eggs, 3–7 days apart. Incubation lasts ~ 39 days, with the nestling period being ~ 86 days in length^14^. Only the first-hatched chick is provided for, with the second-hatched chick mostly perishing as a result of starvation^12^.

Southern Ground-hornbills are threatened as a result of habitat loss and persecution and are considered Vulnerable globally^15^ and Endangered within South Africa^16^. A National Species Recovery Plan was developed in South Africa for Southern Ground-hornbills in 2011^17^. This plan highlighted several knowledge gaps for the species, one being an understanding of the habitat requirements of these birds, as well as important conservation initiatives that could be implemented, such as active relocations of groups to suitable areas within their historical distribution^17^.

Consequently, we tracked the movements and habitat use of six Southern Ground-hornbill groups within the Kruger National Park (KNP), where more than 50% of the South African population of Southern Ground-hornbills is located^18^. Past research on Southern Ground-hornbills suggests that both group and seasonal effects influence home range sizes and habitat use^19,20^. We investigated the seasonal habitat selectivity and space use (how the selected habitat was used) of Southern Ground-hornbill groups of differing size within the KNP. Specifically, we aimed to determine (1) if Southern Ground-hornbills have any seasonal home range differences across the extent of the KNP, and (2) if Southern Ground-hornbills show any seasonal habitat preferences in the KNP. We then used first-passage time analysis to determine (3) at what scale do Southern Ground-hornbills concentrate their foraging movement, and how this varied with season and between groups. Lastly, we determined (4) if Southern Ground-hornbills’ movement behaviours were linked to habitat types. It is hoped that the results of our research will inform management decisions both within and beyond the borders of protected areas, towards the conservation of this endangered bird.

## Results

### Home range and habitat use

A total of 37,060 GPS locations were obtained for the six Southern Ground-hornbill groups, accounting for 3143 group-days. An additional 8746 fixes (19%) were not recorded as a result of satellite signal acquisition failure or battery malfunction. Home ranges of Southern Ground-hornbills determined using KDE ranged from 2866 to 12,145 ha (mean ± SD = 5974 ± 3058 ha; Table 1). All groups showed restricted ranges during the intensive breeding season months (December to March), with the percentage of the overall home range used varying from 21 to 97% (mean ± SD = 63 ± 27.0%). In the early and late dry seasons, groups expanded their ranges with many groups covering areas larger than the extent of their KDE home ranges (101% to 131%, n = 4).

**Table 1:** Home, breeding and seasonal ranges of Southern Ground-hornbills in Kruger National Park measured using Kernel Density Estimates in hectares with the percentage of the home range used in parentheses.

	Cleveland	Jock	Mangake	Mudzadzene	Ngotso Camp	Shingwedzi
Home range (ha)	5080	3757	2866	7154	12,145	4843
Breeding range (Dec-Mar) (ha)	4931 (97%)	2040 (54%)	615 (21%)	5632 (78%)	10,427 (85%)	1974 (40%)
Early wet	3938 (77%)	3417 (91%)	2176 (76%)	4787 (67%)	12,279 (101%)	5749 (118%)
Late wet	4983 (98%)	1976 (52%)	244 (8%)	6509 (91%)	11,673 (96%)	1113 (23%)
Early dry	5862 (115%)	3626 (96%)	3767 (131%)	7230 (101%)	9748 (80%)	3644 (75%)
Late dry	4927 (96%)	3963 (105%)	3298 (115%)	5437 (76%)	13,212 (108%)	4972 (103%)

Southern Ground-hornbill territories comprised only eight of the 72 land cover habitat types with most groups not having equal access to all types. The eight habitat types defined according to the land cover layer were (1) “permanent water”, (2) “seasonal water”, (5) “dense bush, thicket and tall, dense shrubs” (hereafter referred to as dense bush), (6) “woodland and open bushland” (hereafter referred to as open woodland), (7) “grassland”, (9) “low shrubland: other” (hereafter referred to as low shrubland), (36) “Mine (2) semi-bare” (hereafter referred to as gravel quarry), and (41) “bare (non-vegetated)” (hereafter referred to as bare ground). The Ivlev scores for the preference of these habitat types throughout the wet and dry seasons per Southern Ground-hornbill group are given in Table 2. The Shingwedzi and Cleveland groups were the only groups with access to permanent water, although the Ivlev scores show that these areas were strongly avoided year-round. With seasonal water, the Ivelv’s scores are more mixed with some groups actively avoiding these areas and other groups preferentially selecting these habitats. Although available to all groups year-round, bare ground (which includes roads) was strongly avoided. Only the Jock Southern Ground-hornbill group had access to the gravel quarry area, which they selected for in the late dry and early wet seasons, and strongly avoided for the remainder of the year. Dense bush and low shrubland were mostly used in proportion to their availability, although some seasonal preferences were evident. Open woodland and grassland were generally used by all groups in proportion to their availability.

**Table 2:** Ivlev scores of habitat selectivity of Southern Ground-hornbills in Kruger National Park throughout the wet and dry seasons, with no symbols indicating areas used in proportion to their availability.

Season	Group	Permanent water	Seasonal water	Dense bush	Open woodland	Grassland	Low shrubland	Gravel quarry	Bare Ground
Early wet	Cleveland	− 1.00*	− 0.56*	0.11	− 0.08	0.11	− 1.00*	–	− 0.79*
	Jock	–	–	0.11	− 0.01	− 0.04	− 0.15	0.66^ǂ^	− 1.00*
	Mangake	–	− 1.00*	0.01	–0.03	0.06	− 1.00*	–	− 1.00*
	Mudzadzene	–	− 1.00*	0.08	− 0.06	0.03	0.28^ǂ^	–	− 1.00*
	Ngotso Camp	–	0.14	0.58^ǂ^	− 0.29*	0.02	0.14	–	− 0.47*
	Shingwedzi	− 1.00*	− 0.02	0.31^ǂ^	− 0.23	− 0.22	0.34^ǂ^	–	0.16
Late wet	Cleveland	− 1.00*	− 0.90*	− 0.09	− 0.05	0.19	− 1.00*	–	− 0.58*
	Jock	–	–	0.00	− 0.11	0.14	− 0.20	–1.00*	− 1.00*
	Mangake	–	–	0.15	− 0.11	− 0.11	− 0.13	–	–
	Mudzadzene	–	− 1.00*	− 0.02	− 0.20	0.23	0.70^ǂ^	–	0.61^ǂ^
	Ngotso Camp	–	0.28^ǂ^	0.40^ǂ^	− 0.15	− 0.16	0.26^ǂ^	–	− 0.81*
	Shingwedzi	− 1.00*	0.23	0.16	− 0.14	− 0.23	0.58^ǂ^	–	− 0.27*
Early dry	Cleveland	− 1.00*	− 0.80*	− 0.23	0.05	0.14	− 1.00*	–	− 0.84*
	Jock	–	–	− 0.38*	− 0.09	0.23	− 1.00*	− 1.00*	− 1.00*
	Mangake	–	− 1.00*	0.08	− 0.04	− 0.08	0.19	–	− 1.00*
	Mudzadzene	–	− 1.00*	− 0.22	0.12	− 0.07	0.20	–	− 1.00*
	Ngotso Camp	–	0.28^ǂ^	0.31^ǂ^	0.10	− 0.35*	0.12	–	− 0.33*
	Shingwedzi	− 1.00*	0.53^ǂ^	0.13	− 0.16	− 0.17	0.55^ǂ^	–	0.59^ǂ^
Late dry	Cleveland	− 1.00*	− 1.00*	− 0.33*	0.11	0.07	− 1.00*	–	− 1.00*
	Jock	–	–	− 0.28*	− 0.19	0.27^ǂ^	− 0.35*	0.35^ǂ^	− 1.00*
	Mangake	–	–	0.09	0.01	− 0.27*	0.15	–	–
	Mudzadzene	–	− 1.00*	− 0.01	0.06	− 0.08	− 0.29*	–	− 1.00*
	Ngotso Camp	–	− 0.16	0.30	0.01	− 0.09	0.00	–	− 0.80
	Shingwedzi	− 1.00*	− 0.22	0.08	− 0.01	− 0.18	− 0.02	–	0.05

“– “ indicating areas not available to the particular group; “ǂ” = areas used preferentially in relation to their availability and “*” = areas avoided compared with their availability.

The best linear regression model for seasonal Southern Ground-hornbill home range size (AICc = 157.2; Table 3) showed that seasonal home range sizes differed significantly amongst the six Southern Ground-hornbill groups studied. The amount of low shrubland (*p* < 0.005), and grassland (*p* = 0.014) also influenced seasonal home range size, with home ranges decreasing in size with an increase in the proportion of low shrubland available and increasing in size when home ranges contained a higher proportion of grassland habitat. Although not significantly influencing the seasonal home range size, the percentage of dense thicket and open woodland areas were left in the model, as removing these increased the AICc values.

**Table 3:** Parameter estimates, standard errors and p values for variables in the best model (with the lowest AICc) relating the percentage of available foraging habitat with seasonal home range size.

	Estimate	Std. Error	t value	*p*	Significance
Intercept	− 104.44	84.229	− 1.24	0.23537	
Dense thicket	− 21.767	16.356	− 1.331	0.20453	
Open woodland	18.214	14.603	1.247	0.23276	
Grassland	19.204	6.873	2.794	0.01434	*
Low shrubland	− 36.141	6.08	− 5.944	3.58E−05	***
Jock group	55.093	11.598	4.75	0.00031	***
Mangake group	47.347	8.591	5.511	7.67E−05	***
Mudzadzene group	58.338	12.936	4.51	0.00049	***
Ngotso Camp group	106.849	19.132	5.585	6.73E−05	***
Shingwedzi group	76.676	9.112	8.415	7.57E−07	***

Significance codes: ‘***’0.001 ‘**’0.01 ‘*’0.05 ‘.’0.1.

Woody cover was divided into the percentage area of the seasonal home range per Southern Ground-hornbill group that fell into the following categories, < 25%, 25–50% and 51–75%. The best model explaining the influence of woody cover on seasonal Southern Ground-hornbill home range size included only the 25–50% woody cover category (*p* = 0.004, AICc = 159.8) with the percentage area within the 25–50% woody cover category decreasing as seasonal territory size increased.

### First-passage time analysis

There were no clear patterns in the seasonal mean rv_max_ values for the various Southern Ground-hornbill groups (Table 4). Some groups’ rv_max_ values were relatively constant year-round (e.g. Cleveland) whereas others (e.g. Jock and Mangake) fluctuated seasonally. The mean distances moved in the resting movement category were consistent across seasons, with mean foraging distances being similar in the late wet and early dry seasons, and the late dry and early wet seasons. The mean distances for the relocation movement category (> = seasonal rv_max_) were lowest in the early dry season and highest in the early wet season.

**Table 4:** Mean RVmax values obtained from the 10 trajectories randomly selected per group, and mean distance moved per movement behaviour by Southern Ground-hornbill groups in the Kruger National Park, South Africa. These were identified using first-passage time and hourly location intervals, per season.

	Late wet	Early dry	Late dry	Early wet
Group	Mean Rvmax (m) per group per season—mean (SD)			
Cleveland	507.7 (343.1)	568.6 (299.8)	549.0 (132.4)	533.3 (281.8)
Jock	538.5 (291.9)	337.3 (163.8)	611.8 (318.7)	494.1 (203.2)
Mangake	415.4 (228.1)	517.7 (249.8)	431.4 (244.9)	729.4 (287.7)
Mudzadzene	511.5 (393.8)	549.0 (248.0)	780.4 (306.0)	725.5 (358.7)
Ngotso Camp	523.1 (399.0)	392.2 (149.8)	588.2 (202.3)	670.6 (401.2)
Shingwedzi	580.8 (443.7)	560.8 (323.9)	658.8 (305.2)	545.1 (116.9)

Movement mode	Distance moved (m) per season—mean (SD)			
Resting	46.4 (30.5)	41.1 (32.3)	37.2 (33.2)	38.8 (28.7)
Foraging	294.8 (120.0)	292.6 (119.3)	369.2 (144.8)	371.9 (157.2)
Relocating	908.9 (441.5)	715.9 (325.5)	821.6 (350.9)	1024.4 (538.2)

The results from the multinomial regression (Table 5) showed that time spent within the low shrubland, gravel quarry and bare ground habitats had a higher likelihood of being classed within the relocation category as opposed to the foraging category of Southern Ground-hornbills. Conversely, time spent within the grassland, open woodland and dense bush habitat types more likely belonged to the foraging category as opposed to the relocation category. When comparing the resting behaviour with active foraging, all available habitats were preferentially selected for foraging as opposed to resting.

**Table 5:** Results from the multinomial model for the probability of hourly movements within various habitat types being associated with resting or relocating vs. foraging behaviour for Southern Ground-hornbill groups in Kruger National Park.

Variable	Relocating vs. foraging			Resting vs. foraging		
	*β*	SE	*p*	*β*	SE	*p*
(Intercept)	− 0.0461	0.3051	0.8799	0.780	0.257	0.002
Dense thicket	Resting vs. foraging 0.2827	0.3079	0.3586	− 1.614	0.262	0.000
Open woodland	− 0.2232	0.3055	0.4650	− 2.418	0.259	0.000
Grassland	− 0.1453	0.3065	0.6354	− 2.283	0.262	0.000
Low shrubland	0.0775	0.3108	0.8030	− 2.360	0.277	0.000
Gravel quarry	9.6565	67.0236	0.8854	− 3.185	6.003	0.596
Bare ground	0.0464	0.4289	0.9139	− 3.871	1.054	0.000

The estimated coefficients (*β*) are given with standard errors (SE) and significance levels (*p*).

## Discussion

The decision by an individual to move from one area to another is mediated by a number of factors, such as resource quality and availability, predation risk and local environmental conditions, all of which will influence its survival and reproductive output^1,4^. The challenge for conservationists is understanding how these individual decisions can affect population dynamics, home ranges and ultimately species’ survival^1^.

Home ranges of carnivores should overlap and in some cases envelop those of their prey species. Southern Ground-hornbills feed on a variety of prey, ranging from snakes, rabbits and birds to invertebrates^12,18^. Through tracking Southern Ground-hornbill movements, we were able to show that group home ranges during the early and late dry seasons were larger than in the wet season. As the Southern Ground-hornbill breeding season in South Africa coincides with the warm, wet summer months, prey availability, especially that of invertebrates, is expected to be higher^20,21^, suggesting that individuals would not need to travel as extensively to find sufficient food. Furthermore, in the late dry season, groups used between 76 and 115% of their home ranges. This was likely a result of having to increase their search for food and relaxation of the central place foraging required around the nest during the breeding season.

Previous research on Southern Ground-hornbill home ranges has recorded group densities ranging from one group per 4000 ha (communal areas in Zimbabwe^22^), to one group every 10,000 ha (KNP^14^), with one group in the Limpopo Valley having a home range close to 20,000 ha^21^. These results were obtained by direct observations of active nest sites or using VHF radio transmitters. In our study using GPS data, we showed that home range sizes of Southern Ground-hornbills within KNP vary considerably. Despite this, our results confirmed the findings of Theron et al*.*^21^ and Zoghby et al*.*^20^, demonstrating a restricted and contracted home range during the breeding season, when group movements are concentrated around the nest site (central place foraging). Presumably, breeding success would influence the extent of wet seasonal home range for Southern Ground-hornbills, with groups abandoning their central place foraging behaviour when nests fail. Wyness^19^ reported that of four Southern Ground-hornbill groups studied in the Association of Private Nature Reserves (APNR) adjacent to the KNP, the three that bred successfully in the year of their study showed a breeding season range reduction to between 24–36% of their non-breeding home range. The unsuccessful group used 70% of their home range during this time^19^. Surprisingly, the groups within the KNP did not show such a definitive pattern in home range size reduction associated with breeding success, although all groups that attempted breeding did show a wet seasonal home range reduction. Of the six Southern Ground-hornbill groups monitored in our study, four groups bred successfully, one group’s attempt failed (Ngotso Camp), and the breeding status for the third group (Shingwedzi) was unknown. The groups that bred successfully used 21–97% of their respective home ranges, with the unsuccessful group using 85% of their home range (See Table 1).

Southern Ground-hornbills are known to favour more open habitats for foraging^20,23^. Our results supported this, with groups selecting the open woodland and grassland habitat types year-round, following their availability within the landscape.

Although Southern Ground-hornbill seasonal territory size differed significantly amongst the groups, they all showed a decrease in the amount of low shrubland and an increase in the amount of grassland habitat used with increased territory size. Similarly, as seasonal territory sizes increased, the amount of low-medium woody cover (25–50%) decreased. Thus, when selecting an area for a reintroduction of Southern Ground-hornbill groups, the ratio of low-medium woody cover (low shrubland) to grassland, calculated based on the national land cover datasets available, should be taken into account, as this will likely influence the home range size and the number of groups that could be supported in an area.

Although an understanding of the changes and restrictions in territory size is important for the management of a species, the types of movements adopted within a population will influence the management actions needed for their conservation, such as ensuring connectivity or access to certain resources^1^. Conservation policy and management actions are less effective when interventions do not integrate both the spatial and temporal changes in habitat use and the scale of species movements^1,3^. The results from the first-passage time analysis of Southern Ground-hornbill movements showed that the different groups did not consistently demonstrate seasonal patterns in the scale at which they concentrated their foraging efforts. The mean distances travelled for all trajectory paths, classified as active foraging behaviour, were similar and lower in the late wet and early dry seasons compared with the late dry and early wet seasons. Movement between foraging resource patches or mean relocation distances were highest in the wet season months, with the maximum mean distances travelled during the early wet season and the start of the breeding period. Overall prey abundance for Southern Ground-hornbills is generally higher in the wetter months, resulting in a decrease in relocation distances. Our results support the theory that Southern Ground-hornbill wet season movements are most likely influenced by the need to travel to and from the nest site to provision prey to the incubating female and growing nestling. Once resources closer to the nest are depleted, the distances travelled to access additional habitats and prey would likely increase.

Southern Ground-hornbills seemingly prefer nest sites surrounded by more open woodland habitat^24,25^. Habitat structure and the diversity of habitat types within a 3 km radius around the nest site positively influenced Southern Ground-hornbill nesting success. An increase in the density of woody habitat surrounding the nest site, however, had a negative impact on Southern Ground-hornbill breeding success^24^, possibly owing to decreased foraging opportunities, an increased risk of predation or an increase in foraging effort beyond a value which is beneficial.

Habitat structure will likely promote or inhibit the types of movement that can occur in an area. The results from the multinomial regression (Table 5) indicate that the likelihood of a movement behaviour being classified as “foraging” within the open woodland, grassland and dense thicket habitat types was higher than the behaviour being attributed to “relocating”. This is to be expected for open woodland, and grassland habitats as these are both ideal open foraging habitats for Southern Ground-hornbills^20,23^ and are used year-round in proportion to their availability. Southern Ground-hornbills spend around 70% of their day walking^12^ and have been shown to travel distances of up to 10.6 km in a day^20^. Having to navigate through dense thicket vegetation in an area may increase the amount of time spent there, possibly accounting for why this habitat type is predicted to be used more for “foraging”-type behaviour as opposed to “relocating” behaviour. Travel through areas of low shrubland habitat was considered “relocating” behaviour, suggesting that within this habitat type, it is more profitable for Southern Ground-hornbills to move further, and the corresponding chance of finding food greater, than conducting area-restricted searches and spending longer periods concentrated in one patch.

When comparing movements between habitats allocated to “resting” as opposed to “foraging”, the time spent in all habitats was most likely as a result of “foraging”. As GPS locations were only recorded during the day, switching off at dusk (~ 18h00) when Southern Ground-hornbills would roost for the night, habitat preferences for “resting” movements may not have been recorded. Moreover, during the day, Southern Ground-hornbills may not be actively selecting for specific habitat types in which to roost or rest. They may simply be roosting or resting at a chosen site to escape the midday heat within the habitat type in which they were “foraging” or “relocating”.

We were unable to explore differences in movement relating to specific characteristics of the tagged bird (age, sex, helper versus breeder status, etc.) in our study. However, future research should consider study designs able to account for these potential differences, as García-Jiménez et al*.*^26^ showed that both the breeding season and sex of the individual influence displacement and distance travelled in Pyrenean Bearded Vultures (*Gypaetus barbatus*). They found that all individuals travelled more in the breeding season, with females having greater cumulative and maximum distances regardless of the season.

## Conclusions and management implications

As with many species of conservation concern, movement ecology is integral to management decisions regarding Southern Ground-hornbills. Our results have demonstrated habitat preferences, such as the ratio of low-medium woody cover (low shrubland) to grassland, which influence the home range size, and the number of groups of Southern Ground-hornbills that can be supported in an area. These insights are critical to Southern Ground-hornbill conservation, particularly pertaining to the selection of suitable reintroduction sites for the birds. Similarly, sufficient open woodland and grassland habitats are of primary importance and necessary to support the foraging requirements of Southern Ground-hornbills. To aid with the selection of suitable reintroduction sites, and using the seasonal home ranges of Southern Ground-hornbills in the KNP, we determined that for every 1 ha of grassland habitat, potential reintroduction sites should contain a mean of 6.1 ± 3.07 ha of open woodland and a mean of 0.09 ± 0.13 ha of low shrubland. We showed that Southern Ground-hornbill home ranges differed greatly between seasons, mainly as they encompassed different habitat types within the seasons. Consequently, habitat diversity in a potential reintroduction site should be considered integral to the successful establishment of Southern Ground-hornbills there. We suggest that when considering reintroductions of any threatened species, seasonal movements, habitat selection and habitat diversity be taken into account when selecting potential reintroduction sites.

## Methods

### Study area

This study was conducted within the KNP, South Africa (22–26°S, 30–32°E), which comprises approximately 2 million ha. The park is largely divided longitudinally with more granitic soils in the west and basaltic soils in the east^27^ and receives an average annual rainfall of 350–750 mm^28^. Savanna is the dominant habitat type, with pockets of dense woody vegetation found within the broader grasslands^29^.

### Home range and habitat use

This work was part of a registered research project with SANParks (POTTL988) and was performed in accordance with and approved by the animal ethics committees of SANParks, the University of KwaZulu-Natal and the Endangered Wildlife Trust.

**Figure 1:**
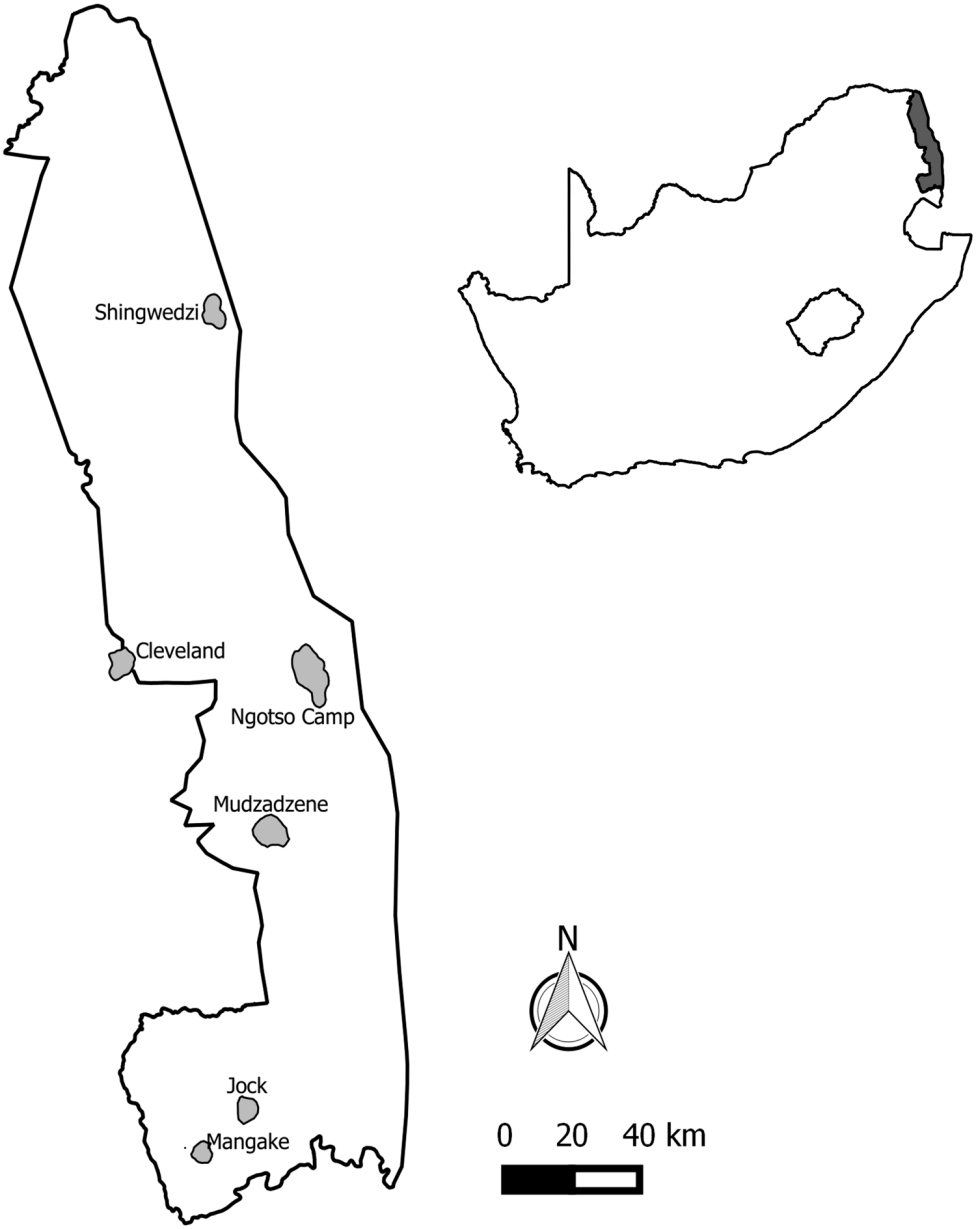
Home ranges of the groups of Southern Ground-hornbills in the Kruger National Park, South Africa tracked during the study period (QGIS version 2.4.0 www.gqis.org ).

We used 70 g solar Argos/GPS PTT satellite transmitters (Microwave Telemetry Inc., Columbia, MD) to track the movements of five groups of Southern Ground-hornbills, four groups within KNP (namely Mangake, Mudzadzene, Shingwedzi and Ngotso Camp) and one group in a conservation area adjacent to KNP (Cleveland) (Fig. 1). An additional KNP group (Jock) was monitored using a 105 g GSM tracking device (VECTRONIC Aerospace GmbH, Berlin, Germany). Groups were named after prominent natural features or infrastructure within their territories. Birds were lured into a domed-shaped walk-in trap (6 m (l) × 3 m (b) × 2 m (h)) using decoy Southern Ground-hornbills made of fibreglass, and simultaneously playing their territorial call. The trap was closed with a curtain which was pulled across the entrance once the birds had entered the trap. One bird per group was fitted with a GPS satellite tracking device using a tubular 16″ Teflon backpack harness design, with the Teflon crisscrossed across the chest and secured onto the back with straps running either side of the bird’s wings. All devices were not programmed according to the same schedule for GPS fixes, as most were donated to the Ground-hornbill project following the termination of other respective tracking projects. Tracking data were prepared for trajectory analysis as per the instructions in the adehabitatLT package^30^, accounting for missing fixes and irregular time intervals.

GPS fixes from the Microwave Telemetry, Inc. (MTI) devices were decoded using their MTI Argos-GPS Parser software, whereas the data from the VECTRONIC Aerospace GmbH (V-A) device used the V-A GPS-Plus software package for GPS data extraction. All statistical analyses were conducted in R version 3.3.1^31^. GIS analyses were conducted using Quantum GIS (QGIS) version 2.4.0^32^, and the R package adehabitatHR^30^ and adehabitatHS^30^. Kernel use density estimates (KDEs) (95%) were calculated using the h_ref_ smoothing factor for the overall home, breeding season and seasonal home ranges using the adehabitatHR package^2,30^. Habitat use was determined using a combination of the Points in Polygon plugin for QGIS and the areas of used and available habitat types within these KDEs.

Habitat preference was determined using a habitat selectivity index (*E*_*i*_), calculated per tracked Southern Ground-hornbill. We used Jacobs’s modification of Ivlev’s Electivity Index^33^, comparing the availability of food types and their utilisation in the diet, modified to reflect habitat preference^34^, according to the following formula

$$ Ei = \left( {pi - qi} \right) / \left( {pi + qi - 2piqi} \right) $$

where *p*_*i*_ = number of satellite fixes per habitat type *(N*_*i*_*)/*Total number of satellite fixes *(N*_*t*_*);* and *q*_*i*_ = area *(ha)* of habitat type in home range *(A*_*i*_*)/*Total area (ha) of home range *(A*_*t*_*).*

The results of this index (*Ei)* range from -1 to + 1, with values >  + 0.25 taken to indicate habitat preference and < − 0.25 taken as habitat avoidance. Values of > − 0.25 to <  + 0.25 indicate neutral habitat attraction. Habitat was classified according to the 2013–2014 South African National Land Cover Dataset^35^. For each KDE, the proportions of the various land types within the KDE were calculated by clipping the land cover layer with the KDE polygons for each Southern Ground-hornbill group generated from the adehabitatHR package^30^. Southern Ground-hornbills coincide their breeding season with the onset of the rainy season, which, in the KNP, falls within the austral summer (November to February). For our study, home ranges were estimated for four seasons: late wet (January to March), early dry (April to June), late dry (July to September) and early wet (October to December).

For each GPS location, the corresponding habitat type was digitally extracted from the land cover layer. The GPS locations contained within the various home and seasonal ranges for each Southern Ground-hornbill group were clipped with the respective polygons and the number of points per habitat type, summed. We also calculated the percentage of woody cover associated with each GPS location. Only a section of the territory of the Cleveland Southern Ground-hornbill group fell within KNP. As the woody cover layer does not extend beyond the KNP boundary, the percentage of woody cover within the Cleveland home ranges could not be calculated, necessitating excluding this group from any analyses involving woody cover.

To determine whether compositional foraging habitat proportions, woody cover, season and group had any effect on the seasonal home ranges of Southern Ground-hornbills, we applied linear regressions to the data using the lme4 package in R^36^. All variables included in the models were log-transformed, and their distributions then approached normality. Separate models for woody cover and habitat composition were run owing to there being no woody cover data for the Cleveland group as mentioned.

### First-passage time analysis

We followed the methods of Fauchald and Tveraa^9^ and used first-passage time (FPT) analyses to determine whether Southern Ground-hornbills exhibit area-restricted search (ARS) behaviour. This analysis places circles of a specified range of radii on the GPS location and calculates the time the particular animal takes to traverse to the centre of the circle and back. Using the adehabitatLT package^30^ for R, we plotted the variance of the log-transformed FPT of ten daily seasonal trajectories per Southern Ground-hornbill group, as a function of the radius. The scale at which the birds concentrated their search (ARS behaviour) corresponded to the value of the radius associated with the peak of the variance of each log-transformed FPT graph9, hereafter referred to as rv_max_. We used the mean rv_max_ for each group per season to determine movement behaviour patterns between successive GPS fixes. We classed movement behaviour as “resting” if the distance between fixes was < 100 m, “foraging” if the distance was ≥ 100 m but < seasonal rv_max_ for the group, or as “relocating” if the distance moved was ≥ the seasonal rv_max_ value. We then used multinomial logistic regression (R package nnet^37^), with “foraging” included as the reference category, to determine the effects of habitat type on Southern Ground-hornbill movement behaviour.

## Data Availability

The GPS-tracking dataset generated and analyzed during the current study is available from the corresponding author on reasonable request.
